# Myocardial Fibrosis in a Recovered COVID-19 Patient: A Case Indicating Prior Myocarditis

**DOI:** 10.7759/cureus.83927

**Published:** 2025-05-11

**Authors:** Ugnė Janonytė, Sofija Goda Cieškaitė, Milda Sėlenytė, Gabrielė Žebrauskaitė-Keblikienė, Tomas Lapinskas, Diana Žaliaduonytė

**Affiliations:** 1 Faculty of Medicine, Lithuanian University of Health Sciences, Kaunas, LTU; 2 Department of Cardiology, Hospital of Lithuanian University of Health Sciences Kauno klinikos, Kaunas, LTU

**Keywords:** coronavirus disease, covid-19, myocardial fibrosis, myocarditis, sars-cov-2

## Abstract

We present a case of a 46-year-old man who was referred to the cardiology clinic for evaluation due to complaints of elevated blood pressure, dyspnea, and chest pain during physical activity for the past four months. The patient had coronavirus disease (COVID-19) five months ago. After the infection, he felt persistent fatigue, restless sleep, and difficulty breathing during physical activity for several months, but this condition gradually improved. Transthoracic echocardiography and magnetic resonance imaging were performed. Late gadolinium-enhanced imaging revealed subepicardial fibrosis limited to the basal and mid segments of the inferior and posterior walls of the left ventricle. This enhancement pattern was interpreted as non-ischemic in aetiology, suggestive of prior myocarditis, potentially related to COVID-19 infection. The patient was started on antihypertensive treatment, and as per the last follow-up, the patient’s blood pressure remained well-controlled, with no other complaints. This case highlights the potential myocardial damage in COVID-19 survivors, highlighting the importance of post-viral cardiac assessment.

## Introduction

Coronavirus disease (COVID-19) has been linked to several cardiovascular manifestations, with a reported 22% prevalence of cardiac injury in hospitalised COVID-19 patients [1,2]. The association between COVID-19 infection and myocarditis has been well-established, with the risk significantly higher compared to other viral respiratory infections [3]. SARS-CoV-2-induced inflammation and coagulation dysfunction lead to cardiomyocyte death, which subsequently promotes myocardial fibrosis [4,5]. Myocardial fibrosis has been observed in patients who were infected with the SARS-CoV-2 virus [6-8]. According to a cohort study of 78 recovered COVID-19 patients, abnormal MRI findings were detected in 78%, with elevated native T1 (n = 73), elevated T2 (n = 60), late gadolinium enhancement (n = 32), and pericardial enhancement (n = 22) [9]. 

We report a case of a 46-year-old male who presented to our outpatient cardiology clinic with complaints of dyspnea and chest pain during moderate physical activity months after recovering from COVID-19. Cardiac imaging revealed subepicardial fibrosis, which was interpreted as non-ischemic in aetiology, suggestive of prior myocarditis, potentially related to COVID-19 infection.

## Case presentation

A 46-year-old male presented to our outpatient cardiology clinic with complaints of dyspnea and non-radiating stabbing chest pain during moderate physical activity. These episodes have been occurring approximately twice daily over four months. They typically resolve spontaneously or after cessation of physical activity and last approximately 2 to 5 minutes. The patient also mentioned having elevated nighttime blood pressure, reaching 160/95 mmHg. Physical examination, including cardiovascular assessment, revealed no abnormalities. The patient has a four-year history of mixed dyslipidemia and arterial hypertension, treated with a fixed-dose combination of rosuvastatin 10 mg and valsartan 160 mg once daily. No other chronic diseases, harmful habits such as smoking, or known drug allergies were noted. From family history, it is known that his father had undergone coronary artery bypass grafting at the age of 55. 

A significant finding is that the patient was infected with COVID-19 five months ago. During this period, he had a fever of up to 38°C and was treated conservatively at home with antipyretic drugs. After the infection, he felt persistent fatigue, restless sleep, and difficulty breathing during physical activity for several months, but this condition gradually improved. 

**Figure 1:**
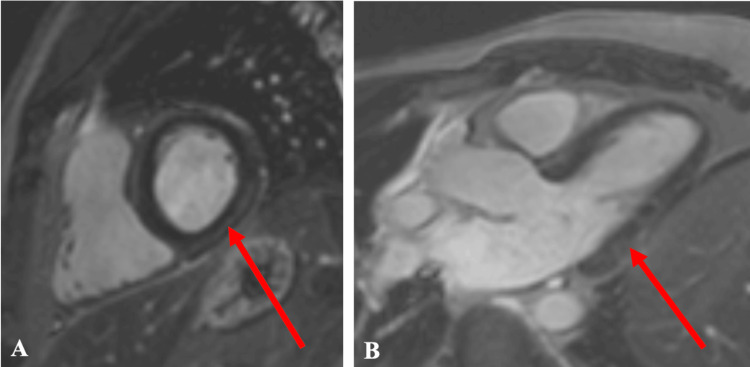
Cardiac magnetic resonance imaging (A-B). Late gadolinium-enhanced imaging reveals subepicardial fibrosis (indicated by red arrows) confined to the basal and mid segments of the inferior and posterior walls of the left ventricle.

Transthoracic echocardiography (TTE) was performed, showing concentric left ventricle (LV) hypertrophy with preserved systolic function and mild diastolic dysfunction. Minimal aortic and mitral regurgitation were also noted. No significant LV outflow tract obstruction or right heart dilation was observed. Pulmonary hypertension (mean pulmonary artery pressure 31.5 mmHg) was observed, and the pericardium appeared normal. Further examination using cardiac 3 Tesla magnetic resonance imaging (3T MRI) showed normal left and right ventricular volumes and ejection fractions. There were no signs of regional wall motion disorders or hypertrophy of the ventricles. T1 and T2 myocardial mapping values were normal. However, late gadolinium-enhanced (LGE) imaging did show subepicardial fibrosis limited to the basal and mid segments of the inferior and posterior walls of the LV (Figure 1). This enhancement pattern was interpreted as non-ischemic in aetiology, suggestive of prior myocarditis, potentially related to COVID-19 infection. Of course, given the absence of any prior history of myocarditis, the current findings could also be attributed to persistent myocardial damage related to COVID-19. 

The patient was prescribed an antihypertensive treatment with a combination of olmesartan 20 mg and hydrochlorothiazide 12.5 mg in the morning and olmesartan 20 mg in the evening. He was also instructed to take 1 mg of rilmenidine if his blood pressure exceeded 140/90 mmHg. Lipid-lowering treatment using rosuvastatin and valsartan was continued. At the last follow-up, two months into this treatment plan, the patient’s blood pressure remained well-controlled, with no other complaints. It was thus further established that the clinical picture and imaging findings matched post-viral myocarditis following COVID-19 infection.

## Discussion

Prevalence of cardiac injury in COVID-19 patients

COVID-19 is known to have a wide range of cardiovascular complications, including myocarditis, type 1 and 2 myocardial infarction, arrhythmias, and other related conditions [1]. A 2021 meta-analysis reported a 22% prevalence of cardiac injury in hospitalised COVID-19 patients. This figure increased to 28% in those with severe disease and was noted to be 30% in patients over 60 [2]. Moreover, there is evidence that the post-acute and chronic phases of COVID-19 infection are also associated with cardiovascular manifestations [10], as meta-analysis findings indicate that 15% of post-acute COVID-19 patients experience cardiovascular sequelae [11]. The association between COVID-19 infection, caused by the SARS-CoV-2 virus, and an increased risk of myocarditis has been previously described in the literature [12], with more recent studies suggesting that this risk is significantly higher compared to other viral respiratory infections. For example, a retrospective cohort study published in 2024 determined that the myocarditis rate per 1,000 person-years was 0.73 for adult COVID-19 patients and 0.24 for adult influenza populations [3]. A prevalence of 2.4 per 1000 hospitalisations is thought to represent the definite or probable acute myocarditis among COVID-19 patients [13]. Interestingly, even recovered COVID-19 patients present an increased risk of myocarditis within one year of the infection [14], proving that myocarditis may manifest in both acute COVID-19 and post-acute sequelae of COVID-19 (PASC) [15]. 

Overview of COVID-19 myocarditis

The clinical presentation typical for myocarditis associated with acute COVID-19 infection is a viral prodrome beginning with symptoms of fever, cough, and shortness of breath, followed by chest pain, symptoms of heart failure, or cardiac arrhythmias [16-18]. Similarly, post-COVID myocarditis can range from mild symptoms like fatigue, chest pain, and shortness of breath to severe cases including arrhythmias, cardiogenic shock, or sudden cardiac death. Typically, myocarditis occurs after the acute phase of COVID-19, with no respiratory symptoms present at diagnosis [19]. Thus, the clinical presentation of COVID-19 myocarditis is hard to characterise and distinguish from other cardiological conditions, as some patients may have a silent presentation or relatively mild symptoms, as was described in our case. In COVID-19 patients, myocarditis is diagnosed using the same criteria as in the general population [20]. The diagnosis is made through laboratory tests, electrocardiogram (ECG), and imaging studies. Inflammatory markers, troponin, and NT-proBNP are often elevated in COVID-19-related myocarditis [21]. ECG abnormalities may be present but are not sensitive enough to detect the disease [17]. Endomyocardial biopsy (EMB) is rarely done due to the invasive nature of the procedure [17]. TTE is recommended to assess ejection fraction, pericardial effusion, and cardiac kinetics, as the findings typically include reduced LV ejection fraction, segmental wall motion abnormalities, increased LV wall thickness, or pericardial effusion [16-17]. Cardiovascular MRI has the highest sensitivity, with results interpreted according to the revised Lake Louise criteria: (i) edema, (ii) irreversible cell injury, and (iii) hyperemia or capillary leak [21]. Imaging findings tend to include impaired LV ejection fraction, pericardial enhancement, diffuse myocardial edema seen on T1 and T2, LGE, subendocardial oedema and enhancement [22]. The treatment of myocarditis in stable COVID-19 patients should be based on standard guidelines, with intravenous corticosteroids and immunoglobulin therapy, reserved for hemodynamically compromised or hyper-inflammatory state patients [17]. 

Pathophysiology of cardiac injury and myocardial fibrosis in COVID-19

SARS-CoV-2 virus causes damage in two stages: (i) viral replication, which leads to direct tissue damage, and (ii) uncontrolled inflammation and cytokine storm. This inflammation, driven by pro-inflammatory cytokines (interleukin-6 (IL-6), IL-1, IL-2, IL-10, tumour necrosis factor alpha, interferon-γ) and coagulation dysfunction, is thought to be the main cause of COVID-19-associated myocarditis [4]. Proving this theory, a case study revealed that an EMB from a COVID-19 patient exhibited myocardial inflammation and viral particles, suggesting a viraemic phase or macrophage migration from the lung [23]. Myocardial fibrosis occurs due to SARS-CoV-2-induced cardiomyocyte death, leading to activation of cardiac fibroblasts, fibrotic response, and deposition of collagen. Nevertheless, the mechanisms of myocardial fibrosis in COVID-19 are not well understood; currently, there are no specific treatment regimens for these patients. Some authors hypothesise that drugs with anti-fibrotic effects (remdesivir, lisinopril, and telmisartan) may have a potential positive therapeutic effect [5]. 

Myocardial fibrosis in COVID-19 patients

We believe that the subepicardial fibrosis observed in 3T MRI images indicates chronic or post-inflammatory changes in the heart muscle, which could present after a viral infection, such as COVID-19, even if the active inflammation has subsided. This idea is supported by previously published studies, where myocardial fibrosis was documented in patients who had been infected with the SARS-CoV-2 virus [6-8]. For example, when evaluating the extent of cardiac manifestations six months after hospital discharge in patients with COVID-19 disease, LGE reflecting myocardial fibrosis was increased in eight patients (21%), of which three had myocarditis [8]. In addition, COVID-19-related myocarditis has been associated with replacement fibrosis, characterised by patchy LGE, often located in subepicardial and mid-myocardial layers [24]. In a retrospective study of 26 post-COVID-19 patients with cardiac symptoms, 58% had abnormal findings on MRI; myocardial edema was seen in 54% and LGE in 31% [25]. Similarly, a cohort study of 78 recovered COVID-19 patients presented abnormal MRI findings in 78%, with elevated native T1 (n = 73), elevated T2 (n = 60), LGE (n = 32), and pericardial enhancement (n = 22) [9]. Jagia et al. highlight three aspects of myocardial fibrosis in patients recovered from COVID-19: (i) myocardial fibrosis may occur without active inflammation; (ii) myocardial tissue abnormalities can precede functional impairment; (iii) LGE predicts poor outcomes in myocarditis, but its prognostic value in COVID-19 is uncertain [26]. 

## Conclusions

This case highlights the potential cardiovascular complications in recovered COVID-19 patients. Subepicardial fibrosis observed on cardiac MRI helps to underline how SARS-CoV-2 may induce persistent structural changes within the myocardium, most probably following an episode of viral myocarditis. Such fibrotic changes might remain clinically silent or present as non-specific manifestations such as chest pain or dyspnea. Since myocardial fibrosis has relevant prognostic implications, in terms of both risk for arrhythmias and potential advancement to heart failure, routine cardiovascular surveillance and imaging should be contemplated in specific post-COVID-19 patients.

## References

[1] Hatab T, Moumneh MB, Akkawi AR, Ghazal M, Alam SE, Refaat MM (2022). COVID-19: cardiovascular manifestations-a review of the cardiac effects. J Geriatr Cardiol.

[2] Fu L, Liu X, Su Y, Ma J, Hong K (2021). Prevalence and impact of cardiac injury on COVID-19: a systematic review and meta-analysis. Clin Cardiol.

[3] Butler O, Raisi-Estabragh Z, Han Y (2024). Epidemiology of myocarditis following COVID-19 or influenza and use of diagnostic assessments. Open Heart.

[4] Shu H, Zhao C, Wang DW (2023). Understanding COVID-19-related myocarditis: pathophysiology, diagnosis, and treatment strategies. Cardiol Plus.

[5] Wang Z, Li L, Yang S (2024). Possible mechanisms of SARS-CoV-2-associated myocardial fibrosis: reflections in the post-pandemic era. Front Microbiol.

[6] Morrow AJ, Sykes R, McIntosh A (2022). A multisystem, cardio-renal investigation of post-COVID-19 illness. Nat Med.

[7] Gorecka M, Jex N, Thirunavukarasu S (2022). Cardiovascular magnetic resonance imaging and spectroscopy in clinical long-COVID-19 syndrome: a prospective case-control study. J Cardiovasc Magn Reson.

[8] Raafs AG, Ghossein MA, Brandt Y (2022). Cardiovascular outcome 6 months after severe coronavirus disease 2019 infection. J Hypertens.

[9] Puntmann VO, Carerj ML, Wieters I (2020). Outcomes of cardiovascular magnetic resonance imaging in patients recently recovered from coronavirus disease 2019 (COVID-19). JAMA Cardiol.

[10] Mohammad KO, Lin A, Rodriguez JB (2022). Cardiac manifestations of post-acute COVID-19 infection. Curr Cardiol Rep.

[11] Huang LW, Li HM, He B, Wang XB, Zhang QZ, Peng WX (2025). Prevalence of cardiovascular symptoms in post-acute COVID-19 syndrome: a meta-analysis. BMC Med.

[12] Xie Y, Xu E, Bowe B, Al-Aly Z (2022). Long-term cardiovascular outcomes of COVID-19. Nat Med.

[13] Ammirati E, Lupi L, Palazzini M (2022). Prevalence, characteristics, and outcomes of COVID-19-associated acute myocarditis. Circulation.

[14] Zuin M, Rigatelli G, Bilato C, Porcari A, Merlo M, Roncon L, Sinagra G (2023). One-year risk of myocarditis after COVID-19 infection: a systematic review and meta-analysis. Can J Cardiol.

[15] Sewanan LR, Clerkin KJ, Tucker NR, Tsai EJ (2023). How does COVID-19 affect the heart?. Curr Cardiol Rep.

[16] Okor I, Bob-Manuel T, Price J (2022). COVID-19 myocarditis: an emerging clinical conundrum. Curr Probl Cardiol.

[17] Cersosimo A, Di Pasquale M, Arabia G, Metra M, Vizzardi E (2024). COVID myocarditis: a review of the literature. Monaldi Arch Chest Dis.

[18] Lovell JP, Čiháková D, Gilotra NA (2022). COVID-19 and myocarditis: review of clinical presentations, pathogenesis and management. Heart Int.

[19] Sayegh MN, Goins AE, Hall MA, Shin YM (2023). Presentations, diagnosis, and treatment of post-COVID viral myocarditis in the inpatient setting: a narrative review. Cureus.

[20] Mele D, Flamigni F, Rapezzi C, Ferrari R (2021). Myocarditis in COVID-19 patients: current problems. Intern Emerg Med.

[21] Siripanthong B, Nazarian S, Muser D (2020). Recognizing COVID-19-related myocarditis: the possible pathophysiology and proposed guideline for diagnosis and management. Heart Rhythm.

[22] Fatmi SS, Basso R, Liaqat A, Tariq F, Swamiappan R (2021). COVID-19 myocarditis: rationale for early diagnosis and intervention. Cureus.

[23] Tavazzi G, Pellegrini C, Maurelli M (2020). Myocardial localization of coronavirus in COVID-19 cardiogenic shock. Eur J Heart Fail.

[24] Rezaeian N, Hosseini L, Asadian S (2021). Cardiac magnetic resonance findings in coronavirus disease 2019. Clin Case Rep.

[25] Huang L, Zhao P, Tang D (2020). Cardiac involvement in patients recovered from COVID-2019 identified using magnetic resonance imaging. JACC Cardiovasc Imaging.

[26] Jagia P, Ojha V, Naik N, Sharma S (2020). Myocardial fibrosis detected by cardiovascular magnetic resonance in absence of myocardial oedema in a patient recovered from COVID-19. BMJ Case Rep.

